# Training of Manual Actions Improves Language Understanding of Semantically Related Action Sentences

**DOI:** 10.3389/fpsyg.2012.00547

**Published:** 2012-12-10

**Authors:** Matteo Locatelli, Roberto Gatti, Marco Tettamanti

**Affiliations:** ^1^Laboratory of Movement Analysis, Vita-Salute San Raffaele UniversityMilano, Italy; ^2^Division of Neuroscience, San Raffaele Scientific InstituteMilano, Italy; ^3^Department of Nuclear Medicine, San Raffaele Scientific InstituteMilano, Italy

**Keywords:** embodied cognition, conceptual-semantics, language understanding, sensory-motor system, action training

## Abstract

Conceptual knowledge accessed by language may involve the reactivation of the associated primary sensory-motor processes. Whether these embodied representations are indeed constitutive to conceptual knowledge is hotly debated, particularly since direct evidence that sensory-motor expertise can improve conceptual processing is scarce. In this study, we sought for this crucial piece of evidence, by training naive healthy subjects to perform complex manual actions and by measuring, before and after training, their performance in a semantic language task. Nineteen participants engaged in 3 weeks of motor training. Each participant was trained in three complex manual actions (e.g., origami). Before and after the training period, each subject underwent a series of manual dexterity tests and a semantic language task. The latter consisted of a sentence-picture semantic congruency judgment task, with 6 target congruent sentence-picture pairs (semantically related to the trained manual actions), 6 non-target congruent pairs (semantically unrelated), and 12 filler incongruent pairs. Manual action training induced a significant improvement in all manual dexterity tests, demonstrating the successful acquisition of sensory-motor expertise. In the semantic language task, the reaction times (RTs) to both target and non-target congruent sentence-picture pairs decreased after action training, indicating a more efficient conceptual-semantic processing. Noteworthy, the RTs for target pairs decreased more than those for non-target pairs, as indicated by the 2 × 2 interaction. These results were confirmed when controlling for the potential bias of increased frequency of use of target lexical items during manual training. The results of the present study suggest that sensory-motor expertise gained by training of specific manual actions can lead to an improvement of cognitive-linguistic skills related to the specific conceptual-semantic domain associated to the trained actions.

## Introduction

Traditional accounts of word meaning have been dominated by work in historical linguistics, mainly dating from the nineteenth and twentieth centuries, promoting a view of lexical-semantic entries as tokens of knowledge shared among speakers of a given tongue that can be derived etymologically and compositionally, as can be described for instance in dictionaries. While this body of work has enormous implications for our formal education and for our daily life in a more and more multilinguistic social environment, the scientific advancement over the last few decades, particularly in the cognitive neurosciences, has emphasized the overwhelming complexity of the brain mechanisms that produce and capture word meaning. Such a major advancement has prompted a need to revise the theoretical accounts of word meanings as relatively crystallized entities in our mind, by taking into account the remarkably plastic and experience-dependent processes undergoing in our brain. One of the most implication-rich aspects of this shift has determined a re-framing in neuroscientific terms of the long-standing dispute among empiricist and rationalist philosophers, beginning from Aristotle as opposed to Plato: in particular, the contemporary neuroscientific dispute has hinged on conflicting views, as to whether lexical-semantic information is represented in our brain in ways that are largely independent from the sensory-motor brain systems, being stored in hetero-modal cortices in an amodal format, or whether on the contrary it derives from sensory-motor experience and as such is deeply rooted in neural networks extending into the sensory-motor system (for recent reviews, see Kiefer and Pulvermüller, 2012; Meteyard et al., 2012).

In the latter view, the retrieval and processing of conceptual knowledge expressed by language in the form of words or sentences re-activates the same primary sensory and motor processes that are involved in the sensory-motor experience of the concepts’ referents. The role of bodily perception and enactment in cognition, including language processing, has been emphasized by proponents of embodiment brain mechanisms (Pfeifer and Scheier, 2001), such as Embodied and Grounded theories focusing either on simulation processes (Barsalou, 1999, 2008), bodily states (Gallese and Lakoff, 2005), or actions situated in a social and physical environment (Glenberg and Kaschak, 2002; Rizzolatti and Craighero, 2004). Empirical evidence has convincingly demonstrated that word meaning is stored in distributed neural networks connecting conceptual content-specific sensory, motor, and emotion-related brain regions with the amodal Perisylvian cortex, with an essential contribution of the left anterior temporal lobe, acting as either a semantic (Patterson et al., 2007) or a modulatory hub (Kiefer and Pulvermüller, 2012). The reactivation of the different neural nodes constituting these distributed semantic networks appears to vary in a highly flexible manner, depending on the type of concept retrieval that is required by the given task, by the context in which it occurs, and by the focus on specific sensory-motor features (Hoenig et al., 2008; Ghio and Tettamanti, 2010; van Dam et al., 2012a,b).

Since the processing of action-related word meaning and congruent motor actions are thought to be subserved, under the flexible circumstances highlighted above, by partially overlapping neural networks, several experimental studies have compatibly shown that the temporal proximity between language processing and action execution tasks can lead to facilitatory/interference effects. For example, Glenberg and Kaschak (2002) showed that hand movements toward or away from the body were facilitated by sentences describing a congruent action (e.g., “He opened/closed the drawer,” respectively), compared to when the hand movement was incongruent with the sentence. Similarly, Zwaan and Taylor (2006) found that sentences describing manual rotation (e.g., “He turned down/up the volume”) facilitated the manual rotation of a knob (to the left/right, respectively). These modulatory effects can occur bi-directionally, as indicated by the finding that, in turn, manual rotation of the knob facilitated reading of sentences that implied a congruent rotation (Zwaan and Taylor, 2006). Boulenger et al. (2006) found that the processing of action-related verbs presented before the signal prompting for an upper-limb grasping movement facilitated movement kinematics, an effect that was ascribed to residual activation of motor areas by verb processing which lowered the amount of activation required by the subsequent grasping movement to reach threshold. In turn, when the action-related verbs were presented simultaneously to the start of the grasping movement, an interference on kinematic parameters was observed; this interference effect was ascribed to language and action processing simultaneously competing for the same neural resources (Boulenger et al., 2006; see also Chersi et al., 2010 for a computational model accounting for these results). Another critical factor for observing a facilitatory effect of action-related sentences onto a subsequent congruent response movement is that the action required for response (e.g., movement toward or away from the body) must have already been known and planned before the onset of sentence processing, as indicated by a study of Borreggine and Kaschak (2006). If, in turn, the required response action is declared to the subjects after sentence processing, the facilitatory effect disappears. This is most likely due to the temporal unavailability of the motor planning system being already engaged in binding other action features (Hommel et al., 2001), which, in the case of action-language compatibility studies, are expressed by action-related sentences (see also Scorolli et al., 2009 for a related finding). Interestingly, this temporal conflict can also arise as an effect of processing two action-related sentences linked by simultaneity, as expressed by the adverb *while*, as opposed to the adverb *after* (de Vega et al., 2004).

Interference between language and action processing can also arise when the two tasks do not overlap in time, provided that the task that precedes induces endurable effects in the shared neural resources. This has been suggested by a study (Glenberg et al., 2008a) reporting a series of behavioral experiments, in which healthy participants were submitted to a repetitive, 20 min long, upper-limb motor task, consisting in moving beans, one at a time, from one container to another, with a movement either toward (one group of participants) or away from (the other group) the body. Immediately after, the participants made semantic sensibility judgments on a set of sentences in which the dimension of interest was between sentences describing object transfer toward or away from the reader. A significant motor task by language task interaction was found, such that participants responded more slowly to sentences with an object transfer direction matching the direction of the upper-limb action previously carried out. The interaction was found both for sentences with a concrete object (“Mark deals you the cards”) and for sentences with an abstract object (“Ann delegates the responsibilities to you”). This result was interpreted as evidence for a saturation effect, making the motor system less responsive to processing action-related sentence content immediately after repetitive execution of a congruent action.

The rapidly growing amount of studies focusing on embodied language in the recent past has raised a hotly debated controversy in the cognitive neuroscience community as to whether distributed representations in the modality-specific cortices are indeed constitutive to conceptual-semantic language understanding, or just an epiphenomenon such as motor imagery (Mahon and Caramazza, 2008). Even among advocates of embodied language theories, there exist different nuances with respect to the constitutiveness argument, leading to a distinction between weak, moderate, and strong versions of the theory (Kemmerer, 2005; Meteyard et al., 2012). Besides what is regarded as evidence of a correlational nature deriving from functional magnetic resonance imaging (fMRI) experiments (e.g., Hauk et al., 2004; Tettamanti et al., 2005; Aziz-Zadeh et al., 2006; Gonzalez et al., 2006; Moscoso del Prado Martin et al., 2006; Kemmerer et al., 2008; Boulenger et al., 2009; Ghio and Tettamanti, 2010), more conclusive evidence on the necessary role of sensory-motor systems has been sought particularly relying on transcranial magnetic stimulation (TMS) of motor areas (Buccino et al., 2005; Pulvermuller et al., 2005a; Glenberg et al., 2008b; Tremblay et al., 2012) and in patients with lesions in the frontal cortex (Bak et al., 2001; Neininger and Pulvermuller, 2003; Cotelli et al., 2006; Bak and Chandran, 2012). Even with respect to TMS and neuropsychology, however, the available evidence remains controversial. As to the former type of studies, Papeo et al. (2009) showed that, contrary to the view that motor areas are rapidly and automatically activated by action-related language processing (Pulvermuller et al., 2005b), action verb processing induced late (500 ms) but not early (170 or 350 ms) modulatory effects on primary motor area activity. Furthermore, they showed that these modulatory effects were only found in a semantic decision but not in a syllabic task, thus suggesting a non-automatic, post-conceptual role of motor area activations in action-language processing. As to the latter type of studies, Papeo et al. (2010) showed that, in spite of results at the patients’ group level confirming the previously described association between motor action deficits and action-related verb processing difficulties (e.g., Bak et al., 2001), in individual patients these two behavioral measures presented a double dissociation, suggesting that the neural systems for language and actions may be largely independent.

In order to help resolving the constitutiveness argument, a crucial notion is represented by causal influences. One needs not only to demonstrate that the processing of word meaning involves the activation of sensory-motor brain areas, but further more that the degree of such an involvement determines the efficiency of conceptual-semantic language understanding. To be truly convincing, this needs to be demonstrated not only in brain-damaged patients or by local perturbations induced by TMS, but also in an unperturbed healthy brain. A substantial leap forward in this direction has been provided by Beilock et al. (2008) in a combined fMRI and behavioral study, showing that specific sensory-motor expertise can improve the comprehension of related concepts in a semantic language task. In this study, ice-hockey players (possessing both playing and viewing experience) were compared to non-player ice-hockey fans (possessing viewing but not playing experience) and novices (no playing or viewing experience). The authors used reaction times (RTs) as a measure of the speed with which the three participant groups matched both the subject and, implicitly, the action-related verb predicate of a sentence with a picture of an individual performing an action presented immediately after. The sentence-picture pairs could refer to either everyday or ice-hockey actions. Beilock et al. (2008) demonstrated that, whereas the three participant groups did not differ in their performance with everyday actions, they significantly differed with ice-hockey actions, with both ice-hockey players and fans producing faster RTs than novices. Furthermore, regression analyses relating brain activation for passive everyday- and hockey-related sentence listening with the behavioral RTs data demonstrated that increasing ice-hockey experience (players > fans > novices) was positively correlated with higher activation of the left dorsal premotor cortex, a brain region supporting the selection of well-learned action plans.

A potential drawback of the Beilock et al.’s (2008) study is that the correlation between sensory-motor expertise and efficiency of conceptual-semantic language understanding was deduced by comparing populations with *de facto* different sport skills and attitudes, so that it is in principle not possible to univocally ascribe more efficient language comprehension to sensory-motor experience, as opposed to other preselected factors. Furthermore, it is in principle equally possible that the semantic understanding advantage of ice-hockey players and fans over novices does not (solely) derive from higher playing and viewing experience, but rather to the more frequent use of ice-hockey-related words in daily life.

In the present study, we aimed to provide direct and clear-cut evidence that sensory-motor training in an homogeneous healthy population can lead to more efficient conceptual-specific semantic processing, as predicted by the constitutiveness argument. To this purpose, over a period of 3 weeks, we trained naive healthy subjects to perform complex manual actions (e.g., origami, prestidigitation, and tying sailor’s knots). Before and after training, each participant underwent a series of manual dexterity tests [Minnesota Manual Dexterity Test (MMDT) and *ad hoc* tests for the trained manual actions], and a semantic language task. The latter consisted of an adapted version of the sentence-picture semantic congruency task employed by Beilock et al. (2008) and allowed us to measure the speed of conceptual retrieval for sentence meanings that were either semantically related (target items) or unrelated (non-target items) to the trained manual actions. We thus manipulated the two factors Semantic condition (target, non-target) and Training phase (pre, post) in a 2 × 2 factorial design with repeated measures. Our expectations were that gaining sensory-motor experience through a prolonged manual action training would lead to a more efficient conceptual-semantic processing of congruent action-related words, resulting into faster post-training RTs specifically for target sentence-picture pairs.

## Materials and Methods

### Participants

Twenty volunteer subjects took part in the experiment: 10 subjects were randomly assigned to group A and 10 subjects to group B. The data of one participant of group A were discarded, due to poor performance in the semantic language task (67% of correct responses). The mean age of the remaining 19 participants (12 women) was 21.1 ± 1.5 years. All participants were right-handed, native Italian speakers and were students of the Vita-Salute San Raffaele University with comparable educational level (high school certificate). They had normal or corrected to normal visual acuity and had no history of neurological, psychiatric, or orthopedic disorders that could affect training or test performance. In order to ensure optimal motor training, we excluded subjects possessing specific abilities related to the trained manual dexterity tasks (origami folding, tying sailor’s knots, prestidigitation/rolling coins, sewing, and keyboard playing).

All volunteer subjects gave written informed consent to participate after receiving an explanation of the procedures, according to the Declaration of Helsinki, while remaining naive as to the purpose of the study. The study was approved by the Ethics Committee of the San Raffaele Hospital, Milan.

### Manual dexterity training

Each participant trained in three manual dexterity motor tasks. Participants of both groups trained to make origami. Participants in group A also trained to tie sailor’s knots, and to roll coins across their fingers. Participants in group B also trained to sew, and to play finger tapping sequences according to color-coded scores. Thirty-minute long training sessions were scheduled over a period of 3 weeks, 5 days/week, 10 min/task. The total of 15 training sessions for each participant were ordered so as to increase task difficulty over the 3-week training period. Task instructions were provided in the form of either still or silent motion pictures, carefully avoiding any accompanying verbal descriptions, particularly with respect to target action-related verbs.

Origami folding was performed using standard square origami paper. Increasing difficulty was achieved by training a different origami figure in each session, with figure in successive sessions displaying an increasing number of required folds and steps.

Sailor’s knots were tied using two ropes (length: 1 m; diameter: 0.008 m). Increasing difficulty was achieved by training a different knot in each session, with knots in successive sessions displaying an increasing number of required manipulations and loops.

Coins were rolled from the index, to the middle, ring, and finally to the little finger knuckles of the right hand. Increasing difficulty was achieved by reducing the size of the coin every three sessions (2 euro coin, 50 cent euro coin, 1 euro coin, 20 cent euro coin, and 10 cent euro coin).

Sewing was performed with a sewing needle and all-purpose sewing thread. Fabric sheets with printed line drawings were provided. The participants sewed along the line drawings, using a uniform running stitch. Increasing complexity was achieved by training a different line drawing in each session, with drawings in successive sessions displaying an increasing number of elements and segments.

Finger tapping sequences were performed on a sheet of paper with seven printed circles, each circle of a different color. Scores were provided, consisting of sequences of color-number pairs. One pair after the other, the participants tapped the corresponding colored circle with the right hand finger indicated by the associated number (2: index; 3: middle; 4: ring; and 5: little). The finger tapping frequency was set by a metronome. Increasing difficulty was achieved by changing the scores every three sessions, and for each score, by increasing the metronome frequency every session (60, 90, and 120 bpm).

In order to control for verbal descriptions that the participants may have explicitly associated to the different motor tasks, at the end of the training period we asked participants to write descriptions of the manual dexterity tasks that they performed (see last paragraph of Semantic Language Task Data for information on how the written descriptions were scored and employed for the data analysis).

Before and after the training period, the participants were submitted to, respectively, pre-training and post-training manual dexterity assessments and a semantic language task.

### Manual dexterity assessments

The MMDT (Lafayette Instrument Company, Lafayette, IN, USA) was used to assess manual dexterity (Elfant, 1977; Lee and Tsang, 2001). The participants performed three trials of the MMDT turning task (Mandell et al., 1984), using their right hand. The mean of the scores obtained in the last two trials was considered for the analysis.

In addition, in order to specifically evaluate the improvement in performance in the trained manual dexterity motor tasks, we devised a specific metric for each task. For origami, we measured the time employed by each participant to faithfully fold the simplest figure in the training series. For tying sailor’s knots, we measured the time employed by each participant to faithfully tie the simplest knot in the training series. For coin rolling, we measured the number of times (cycles) each participant errorlessly rolled the 2 euro coin from the index to the little finger knuckle during a 1-min interval. For sewing, we asked participants to sew along the simplest drawing in the training series, and counted the number of flawless stitches during a 1-min interval (stitches falling outside the drawing lines were considered as mistakes and not counted). Finally, for sequential finger tapping, we asked participants to tap according to the first score in the training series at a frequency of 60 bpm, and counted the number of mistakes (i.e., wrong colors, wrong fingers, and misses). The finger tapping performances were video-taped for subsequent scoring.

Note that in the pre-training assessments, the participants were confronted for the first time with novel tasks, but this is true both for the standardized MMDT and for the *ad hoc* manual dexterity motor tasks. In order to minimize the influence of procedural over manual novelty from the pre-training to the post-training sessions, participants were given sufficient time (5 min) to familiarize with the task instructions. In addition, each task was performed three times: the first trial served for familiarization, whereas only the last two trials was considered for the analysis by taking their mean score.

### Linguistic stimuli

For each of the two participants’ groups, we selected six manual action-related Italian transitive verbs describing the corresponding trained manual dexterity actions (Table 1). These verbs constituted the target semantic condition, for which we expected a specific facilitation at the conceptual-semantic level induced by manual training. As an experimental control, we also selected for each group six manual action-related transitive verbs, whose meaning was not associated with the trained manual dexterity actions (Table 1). These verbs constituted the non-target semantic condition. Note that, since two out of the three manual actions trained by each group differed between groups A and B, most of the verbs in the target semantic condition for group A could be used as verbs in the non-target semantic condition for group B, and vice versa (Table 1). Thus, the separation of participants in the two groups A and B served as a partial reciprocal control for the specificity of the manual training effect over conceptual-semantic verb processing. In other words, we expected the same verb to be associated both with a conceptual-semantic facilitation in the group where it belonged to the target semantic condition (e.g., group A), and with significantly reduced or no effects in the group where it belonged to the non-target semantic condition (e.g., respectively, group B).

**Table 1:** **List of Italian verbs and English translations**.

Group A		Group B	
Manual action	Verb	Manual action	Verb
**TARGET**			
Origami	Piegare (to fold)	Origami	Piegare (to fold)
Tying knots	Agganciare (to hook)	Sewing	Cucire (to sew)
	Allacciare (to fasten)		Rammendare (to darn)
	Annodare (to tie)		Ricamare (to embroider)
	Infilare (to thread)		Infilare (to thread)
Rolling coins	Manipolare (to handle)	Finger tapping	Digitare (to key in)
**NON-TARGET**			
	Avvitare (to screw)		Avvitare (to screw)
	Disegnare (to draw)		Disegnare (to draw)
	Cucire (to sew)		Agganciare (to hook)
	Digitare (to key in)		Allacciare (to fasten)
	Rammendare (to darn)		Annodare (to tie)
	Ricamare (to embroider)		Manipolare (to handle)
**FILLER**			
	Abbottonare (to button up)		Abbottonare (to button up)
	Accarezzare (to stroke)		Accarezzare (to stroke)
	Grattare (to scratch)		Grattare (to scratch)
	Impugnare (to clasp)		Impugnare (to clasp)
	Iniettare (to inject)		Iniettare (to inject)
	Levigare (to rub down)		Levigare (to rub down)
	Pennellare (to paint)		Pennellare (to paint)
	Pizzicare (to pinch)		Pizzicare (to pinch)
	Ritagliare (to cut out)		Ritagliare (to cut out)
	Sbucciare (to peel)		Sbucciare (to peel)
	Sfogliare (to leaf through)		Sfogliare (to leaf through)
	Sminuzzare (to chop up)		Sminuzzare (to chop up)

The lexical frequency of verbs in the target versus non-target semantic conditions was balanced, using the Italian Corpus of Lexical Frequency (Laudanna et al., 1995), for both group A (*P* = 0.49) and group B verbs (*P* = 0.84). The number of letters (group A: *P* = 0.49; group B: *P* = 0.11) and syllables (group A: *P* = 0.61; group B: *P* = 0.08) was also balanced across the two conditions.

To serve as fillers for the semantic language task, we also selected 12 additional manual action-related transitive verbs, which did not bear any semantic relationships with the manual actions trained by either groups A and B (Table 1).

### Semantic language task

We paired each verb in Table 1 with a color photograph of a manual interaction with objects taken from a standardized set of stimuli, which was developed to investigate the retrieval of lexical and conceptual action knowledge (Fiez and Tranel, 1997). For filler verbs, the manual action depicted in the photograph was incongruent with the verb meaning. For verbs in the target and non-target semantic conditions, it was congruent with the verb meaning. Some congruent color photographs had to be taken *ad hoc*, as the corresponding actions were not present in the Fiez and Tranel set; for this, we used visual conventions matching as closely as possible those of the Fiez and Tranel set. Importantly, the congruent manual action depicted in the congruent photographs did not bear direct resemblance with the actions trained by the participants (e.g., the picture for “to fasten,” associated to tying knots, represented a right and a left hand fastening shoe ties), and in some cases it was largely unrelated (e.g., the picture for “to manipulate,” associated to rolling coins, represented a right and a left hand manipulating modeling clay). This was done in order to eliminate the potential bias in the semantic language task, deriving from visual familiarity with the situation depicted in the photographs, when the latter is similar to the situation experienced during manual dexterity training.

In order to measure the speed of lexical-conceptual retrieval in a semantic language task, we used an adapted version of the task employed by Beilock et al. (2008). All selected verbs were used in the third person singular, present simple tense form to create short declarative sentences of the form “Quella persona disegna” (English: “That person draws”). Participants were presented with one sentence at a time for 1000 ms, followed by a 500-ms interval and by the associated picture for 3000 ms. The required task was to indicate, as quickly as possible, the congruency/incongruency of each sentence-picture pair by hitting the right (congruent) or left (incongruent) arrow keyboard key with the, respectively, middle and index right hand fingers. The 24 sentence-picture pairs were presented consecutively in one single block. Sentence-picture pairs were separated by a variable interval of either 3500, 4000, or 4500 ms. The pairs were presented in semi-randomized order, with different randomizations for the pre-training and the post-training sessions.

Stimulus presentation and response collection was controlled by a laptop with a 17″ monitor, using Psychopy 1.64 software (Peirce, 2009). We calculated RTs as the time elapsed between the onset of picture presentation and the participant’s response. The RTs for filler sentence-picture pairs were not analyzed.

Prior to the experimental sessions, the participants familiarized with the task instructions and performed a short familiarization block with four sentence-picture pairs not included in the experimental set.

### Statistical analysis

A significance α level of 0.05 was declared for all analyses.

#### Manual dexterity assessment data

In order to evaluate the effectiveness of manual training, MMDT scores and the scores relative to the specific metrics for the trained manual dexterity motor tasks were submitted to paired Student’s *t*-tests comparing the post-training with pre-training performance.

#### Semantic language task data

The collected RTs of all participants were pooled over groups A and B, according to the two semantic conditions (target versus non-target) and the two training phases (pre versus post). We discarded the RTs for incorrect answers, as well as those falling 2 SD above or below each subject’s mean.

The effects of manual training onto target and non-target semantic processing were evaluated by means of paired Student’s *t*-tests comparing post-training with pre-training RTs.

In order to investigate whether manual training induced a specific facilitation in the conceptual-semantic processing of target versus non-target stimuli, we used a repeated measures ANOVA on by-subject aggregated data with a 2 × 2 factorial design, the experimental factors being Semantic condition (target, non-target) and Training phase (pre, post). The assumption of sphericity was controlled by means of the Mauchly’s sphericity test. We calculated main effects and interactions. A *post hoc* one-tailed Student’s *t*-test was used to verify that post-training target stimuli were processed faster than post-training non-target stimuli.

A further analysis was performed in order to eliminate the potential bias deriving from the fact that the participants may have explicitly associated verbal descriptions to the trained manual dexterity tasks. We scored the written descriptions provided by the participants at the end of the training period (see Manual Dexterity Training) and eliminated from the analysis all the RTs relative to target sentence-picture pairs containing verbs referred by one or more participants. We then repeated the same 2 × 2 factorial analysis as for the complete stimulus set, including the sphericity test. Two participants of group B were left out from this analysis, as they did not provide any valid responses to the reduced stimulus set.

## Results

### Results of the manual dexterity assessments

We compared the pre-training and post-training scores obtained in the MMDT and in the specific assessments for the trained manual dexterity motor tasks. All tests showed significant training effects in both groups (Table 2).

**Table 2:** **Pre- and post-training motor dexterity assessments**.

	Group A			Group B		
	Pre-training	Post-training	*t*-Test	Pre-training	Post-training	*t*-Test
MMDT	65.7 ± 8.3 s	59.2 ± 7.4 s	*P* = 0.0012	68.0 ± 4.5 s	60.1 ± 4.0 s	*P* = 0.0003
Origami	24.9 ± 5.8 s	11.7 ± 2.4 s	*P* < 0.0001	23.5 ± 5.1 s	14.6 ± 4.2 s	*P* < 0.0001
Knots	35.6 ± 17.2 s	13.0 ± 4.0 s	*P* = 0.0026			
Coins	0.7 ± 0.5 cycles	8.9 ± 2.5 cycles	*P* < 0.0001			
Sewing				7.2 ± 3.3 stitches	16.9 ± 2.1 stitches	*P* < 0.0001
Tapping				10.1 ± 5.2 errors	0.5 ± 0.5 errors	*P* = 0.0002

*Mean scores and SD for each measure pre- and post-training are indicated, together with the significance *P*-value of the paired Student’s *t*-tests comparing the post-training with the pre-training performance*.

### Results of the semantic language task

The participants made on average 88.6% (SD ± 9.3) correct responses in the pre-training session and 92.1% (SD ± 7.1) correct responses in the post-training session, with no significant differences between sessions [*t*(37) = 1.539; *P* = 0.132]. The accuracy did not significantly differ between target and non-target sentence-picture pairs, neither pre-training [*t*(18) = 1.302; *P* = 0.209], nor post-training [*t*(18) = 1.286; *P* = 0.215]. The lack of significant results for accuracy replicates the observations of Beilock et al. (2008) using an almost identical semantic language task, and can be explained by the overall ease of the task. Accordingly, our prior hypotheses spelled out in the Section “Introduction” did not concern accuracy, but only RTs.

For target sentence-picture pairs, the participants responded on average after 652 ms (SD = 120) pre-training and 495 ms (SD = 76) post-training, with a significant [*t*(18) = −5.845; *P* = 0.000007] RTs reduction in the post-training session. A qualitatively similar effect was observed for non-target sentence-picture pairs: the participants responded on average after 633 ms (SD = 124) pre-training and 519 ms (SD = 65) post-training, with a significant [*t*(18) = −4.856; *P* = 0.00006] RTs reduction in the post-training session.

**Figure 1:**
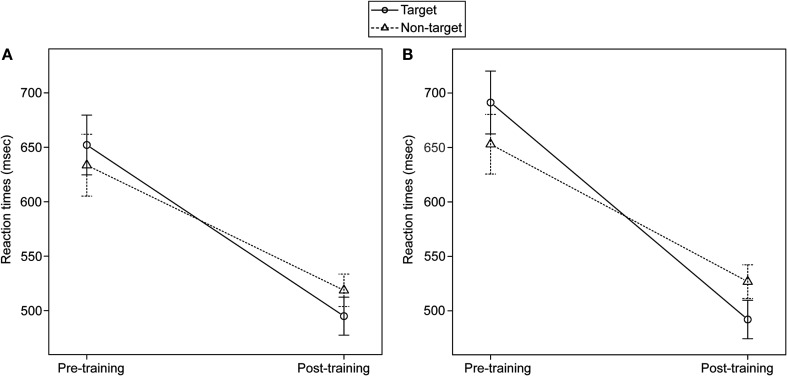
**Semantic condition by Training phase interaction in the semantic language task**. Mean RTs across all participants and SE bars are represented for the four experimental conditions resulting from the 2 × 2 factorial combination of Semantic condition (target, non-target) and Training phase (pre, post). **(A)** Mean RTs for all sentence-picture pairs. **(B)** Mean RTs in the reduced data set, correcting for the potential bias deriving from the explicit verbal descriptions of the trained manual dexterity tasks given by the participants.

As a crucial analysis for our experimental question, we assessed whether manual training induced a specific facilitation in the conceptual-semantic processing of target versus non-target sentence-picture pairs, by using a 2 × 2 repeated measures ANOVA. The main effect of Semantic condition was not significant [*F*(1,18) = 0.091; *P* = 0.767], whereas the main effect of Training phase was significant [*F*(1,18) = 32.683; *P* = 0.00002]. Most importantly, the Semantic condition by Training phase interaction was also significant [*F*(1,18) = 5.953; *P* = 0.025]. Accordingly, the post-training RTs for target sentence-picture pairs were significantly faster than those for non-target sentence-pictures pairs [*t*(18) = −2.242; *P* = 0.019] (Figure 1A).

The participants may have responded faster to target versus non-target sentence-picture pairs after training simply because they explicitly associated verbal descriptions to the trained manual dexterity tasks. To control for this potential bias, we eliminated the responses to stimulus pairs whose action-related verb had been used by one or more participants in their written descriptions of the trained manual dexterity tasks provided after the post-training session. This left us with the responses for stimuli containing, for group A, the target verbs “to hook,” “to fasten,” “to thread,” and “to handle,” and for group B, the target verb “to darn” (confront with Table 1). The mean RT was 691 ms (SD = 119) for target pre-training and 492 ms (SD = 73) for target post-training, with a significant [*t*(16) = −7.023; *P* = 0.000001] RTs reduction in the post-training session. For non-target picture-sentence pairs the mean RT was 653 ms (SD = 113) pre-training and 527 ms (SD = 64) post-training, with a significant [*t*(16) = −5.164; *P* = 0.00005] RTs reduction in the post-training session. The 2 × 2 repeated measures ANOVA with this reduced response data set again showed that the main effect of Semantic condition was not significant [*F*(1,16) = 0.028; *P* = 0.870], that the main effect of Training phase was significant [*F*(1,16) = 47.897; *P* = 0.000003], and, most importantly, that the Semantic condition by Training phase interaction was also significant [*F*(1,16) = 9.006; *P* = 0.008]. Accordingly, the post-training RTs for target sentence-picture pairs were significantly faster than those for non-target sentence-pictures pairs [*t*(16) = −2.557; *P* = 0.011] (Figure 1B).

## Discussion

The processing of action-related word meaning is thought to rely on distributed neural networks involving the amodal Perisylvian cortex and extending to the sensory-motor system, in a manner that flexibly depends on the context and task (Ghio and Tettamanti, 2010; van Dam et al., 2012b; Kiefer and Pulvermüller, 2012). The controversy as to whether the involvement of the content-specific sensory-motor cortices are indeed constitutive to conceptual-semantic language understanding or instead just an epiphenomenon (Mahon and Caramazza, 2008; Meteyard et al., 2012) requires, in order to be further attacked, convincing evidence of causality, demonstrating that the degree of the sensory-motor involvement indeed determines the efficiency of conceptual-semantic language understanding, particularly in the intact, healthy brain. In the present study, we have attempted to fulfill these requirements by training naive healthy subjects to perform complex manual actions over a period of 3 weeks, and by measuring the post- versus pre-training effect on a semantic language task distinguishing between sentence meanings that were either semantically related (target items) or unrelated (non-target items) to the trained manual actions. Consistently with our hypothesis and with the constitutiveness argument, we found a significant Semantic condition by Training phase interaction, and showed that the interaction was accounted for by faster post-training RTs responses specifically for target versus non-target stimuli. This is suggestive of a causal relationship between action-related language processing and sensory-motor brain regions controlling manual actions. Due to the purely behavioral nature of our measurements, we cannot provide here any detailed descriptions of the involved sensory-motor brain regions, but we can speculate based on a previous neuroimaging study (Beilock et al., 2008) that these crucially involve the left dorsal premotor area. More in general, other brain regions of the action representation system distributed in the inferior frontal, parietal, and temporal lobes may also be involved (Grafton and Hamilton, 2007; Ghio and Tettamanti, 2010).

It is important to note that, above and beyond these essential neuroanatomical specifications, the implied causal relationship between action-related language processing and the motor system is not merely of a locationist type, such that shared brain regions become involved, for example, through Hebbian association learning (see, e.g., Fargier et al., 2012), but truly functional. The higher activation and/or neuronal density of relevant components of the motor system that follows from the experience-dependent acquisition of finer action control skills leads to a more efficient semantic comprehension of words or sentences conveying the corresponding concepts, as shown in the present and in the Beilock et al.’s (2008) study. This functional as opposed to a locationist conceptualization of causality also nicely fits with the view that the activation of each neural node constituting a distributed semantic network can be modulated in a flexible manner (Kiefer and Pulvermüller, 2012), with a full involvement of the semantic network producing a most vivid conceptual representation.

Although the concept-specific facilitation effect induced by manual training in the present study could be predicted based both on the constitutiveness argument that the degree of involvement of sensory-motor brain areas determines the efficiency of conceptual-semantic language understanding, and on the previous study by Beilock et al. (2008), there are other circumstances, as described in the Section “Introduction,” in which the shared exploitation of common neural resources by language and action processing leads to interference effects. In particular, Glenberg et al. (2008a) let participants perform a repetitive manual task and found a concept-specific slowing down of RTs in a semantic language task performed immediately after. There were several methodological differences between the present study and the study by Beilock et al. (2008), on the one side, and the study of Glenberg et al. (2008a), on the other side, that may justify this discrepancy, including the fact that in the latter study the participants performed a highly stereotyped movement in one single session of 20 min, which was most likely not challenging enough to lead to an expansion of their sensory-motor experience and repertoire. This is even more likely the case, since the stereotyped movement (moving beans) consisted of a well-learned motor behavior, as opposed to teaching a new behavior as in the present study. The temporal windows of neural plasticity investigated may also play an important role: the saturation effect (of probable neurophysiological origin) observed in the Glenberg et al.’s (2008a) study immediately after a brief motor task session may turn into a facilitation effect if the motor task is protracted over multiple sessions and if a sufficient amount of time elapses between the motor and the language tasks in order to permit structural neural plasticity to develop. This latter, long-term scenario may be more closely related to the experimental setting in both the Beilock et al.’s (2008) study, in which expertise was roughly equated to enduring individual attitudes, and in the present study, in which the participants were trained in complex manual actions over a period of 3 weeks.

The adoption of a long-term sensory-motor training program, protracted over a period of 3 weeks, to investigate plasticity effects on conceptual-specific semantic processing, is a major factor of experimental novelty of the present study, compared to the large amount of previous studies on action-language compatibility effects (e.g., Glenberg and Kaschak, 2002; de Vega et al., 2004; Borreggine and Kaschak, 2006; Boulenger et al., 2006; Zwaan and Taylor, 2006; Scorolli et al., 2009). This type of long-term training paradigms may be particularly helpful in the future to further explore, in an experimentally controlled manner, how conceptual-semantic linguistic representations are dynamically tuned by the constantly changing sensory-motor experiences across the individual life-time, similar to an increasingly widespread approach for cognitive studies outside the language domain (see, e.g., Kiefer et al., 2007; Weisberg et al., 2007; Bellebaum et al., in press).

It is also important to note that, although the Semantic condition by Training phase interaction indicates that there was a specific effect of manual training on target conceptual-semantic processing, the paired comparisons contrasting the post-training versus pre-training performance, separately for target and non-target sentence-picture pairs, showed a marked decrease of RTs for both the target and the non-target conditions. The present study does not allow to distinguish between an interpretation of this general effect as being due either to an unspecific gain of procedural, motor, and executive skills induced by manual dexterity training (such that the participants were simply more responsive and compliant to the task’s requests after training), or to a carry-over effect of increased sensory-motor resources also available for the conceptual-semantic processing of non-target action-related verbs. In the former view, the greatest proportion of the variance of the Training phase effect would be explained by non-semantic factors related to manual dexterity, with conceptual-semantic factors inducing only a relatively smaller gain of response efficiency limited to the target condition, as represented by the Semantic condition by Training phase interaction. In the latter view, manual training conferred more efficiency to the semantic processing of both target and non-target action-related verbs, but with a significantly higher effect specifically for target verbs. This would be possible, for instance, if the neural plasticity effects induced by training in the dorsal premotor cortex would partially propagate from cell populations specific for the trained manual actions to other surrounding cell populations coding for other (non-target) manual actions. It is of course also possible that both factors contributed to the observed generalized effect of Training phase. Further studies will be required to discriminate between these scenarios.

There are in principle a few alternative explanations to account for the results presented here. The first alternative explanation is that the greater post-training RTs reduction observed for target compared to non-target sentence-pictures pairs may not be due to the greater sensory-motor experience acquired through training of the related manual actions, but simply to the fact that during training the participants used the target action-related verbs more frequently (e.g., to describe, rehearse, or plan the trained actions) – a concern that, as noted in the Section “Introduction,” was not accounted for in the Beilock et al.’s (2008) study. While it is not possible to monitor the ongoing lexicon retrieval of the participants during the training period, we have done our best to control for this potential bias, by asking the participants at the end of the training period to provide written verbal descriptions of the trained motor tasks. We scored these descriptions and eliminated all the responses relative to target sentence-picture pairs containing verbs explicitly referred by even just one or by more than one participant. We then submitted this reduced response data set to the same 2 × 2 factorial analysis used for the complete set. The results of this control analysis were qualitatively identical to those of the complete response data set, and the significance levels of both the Semantic condition by Training phase interaction and the *post hoc* comparison between post-training target and non-target sentence-picture pairs were even increased. We are therefore confident that the acquired sensory-motor manual expertise, rather than simply verbal rehearsal, caused the observed concept-specific improvement in the semantic language task.

The second alternative explanation is that the observed results may again not be due to the greater sensory-motor experience acquired through manual training, but rather to a visual familiarity between the situation depicted in the pictures presented in the semantic language task with sentence-picture pairs and the situation experienced during manual training (e.g., objects manipulated, hand posture, and visual angle). However, as noted in Section “Semantic Language Task,” the manual actions depicted in the photographs belonging to the target sentence-picture pairs did not bear direct visual resemblance with the manual actions trained by the participants. For example, the picture for the target verb “to fasten,” associated to the trained manual action “tying knots,” represented a right and a left hand fastening shoe ties; the picture for the target verb “to manipulate,” associated to the trained manual action “rolling coins,” represented a right and a left hand manipulating modeling clay. The crucial notion here regards the separability of visual similarity from conceptual-semantic processing in the context of processing pictures in our semantic congruency judgment task. Neurophysiological studies in monkeys and neuroimaging studies in humans have provided abundant evidence that the visual recognition of an observed action involves two highly integrated but distinct neural pathways: one “dorsal” pathway for the analysis of how the action is physically carried out in relation to, for example, the object’s location, size, and affordances, the hand’s location and haptic configuration, and the required sequence of motor acts; and one “ventral” pathway for analyzing the “abstract” meaning of the observed action (Arbib, 2012). Our effort to minimize the visual resemblance between the depicted and the trained actions was precisely aimed at eliminating as much as possible any effects of visual priming that may result from some overlap of neural coding in the “dorsal” pathway. Considering the example of fastening shoelaces in comparison to the trained action of tying sailor’s knots, there were notable differences with respect to location, size, and affordance of laces versus rope objects, different hand configurations, and a different sequence of motor acts. This in contrast to the shared “abstract” semantic notion of “fastening/tying knots,” which may lead to a neural coding overlap in the “ventral” pathway by both the depicted and the trained action. This latter overlap is however not a matter of concern, as it relates precisely to the conceptual-semantic level that we aimed to assess. We are therefore again confident that the effect of visual similarity also did not bias the language understanding improvement effect.

In sum, we conclude that an increase in sensory-motor expertise gained by training of specific manual actions can lead to a more efficient semantic processing of the specific action-related conceptual domain associated to the trained actions, with a possible, relatively less pronounced, carry-over effect to the entire action-related domain. This modality-specific effect most likely depends on shared neural resources between the sensory-motor system and conceptual-semantic language processing and implies a bidirectional causal link, in which sensory-motor experience can influence word meaning representations and the processing of word meaning can in turn influence sensory-motor representations. This latter aspect may be revealed by future research.

## Author Contributions

Designed research: Matteo Locatelli, Roberto Gatti, and Marco Tettamanti. Performed research: Matteo Locatelli. Analyzed data: Matteo Locatelli and Marco Tettamanti. Wrote the paper: Matteo Locatelli, Roberto Gatti, and Marco Tettamanti.

## Conflict of Interest Statement

The authors declare that the research was conducted in the absence of any commercial or financial relationships that could be construed as a potential conflict of interest.
